# Supplementary material for: Unraveling spatial cellular pattern by computational tissue shuffling
Source: Commun Biol. 2020 Oct 23;3:605. doi: 10.1038/s42003-020-01323-3 (PMC7584651; doi:10.1038/s42003-020-01323-3)
Supplement: Supplementary file 1 — Supplementary Information [file 42003_2020_1323_MOESM1_ESM.pdf]

Supplementary Information

Unraveling spatial cellular pattern by computational tissue shuffling

Supplementary Figures

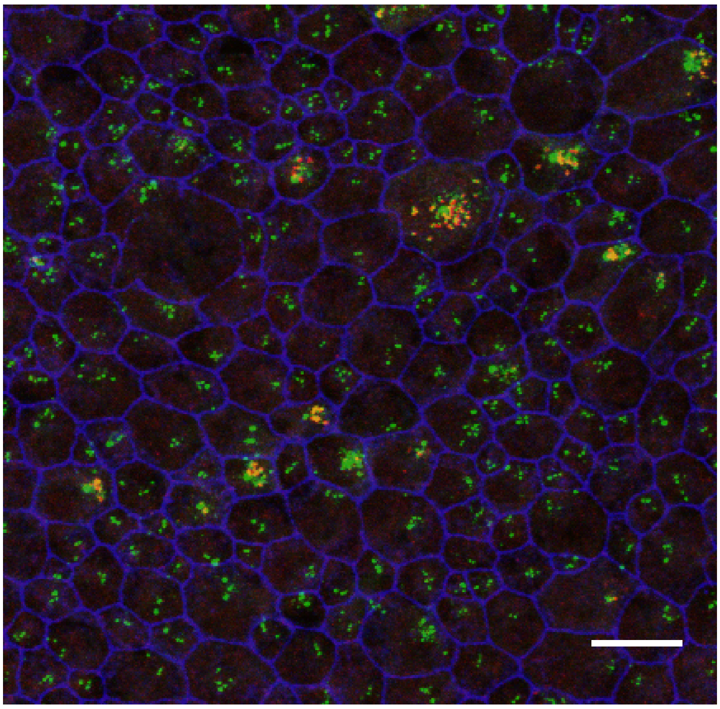

Supplementary figure 1 - P1 mice ependymal tissue. *Cell junctions labeled with ZO1 antibody (blue), centrioles labeled with Centrin2-GFP (green), procentrioles labeled with Sas6 antibody (red). Scale bar: 10 µm.*

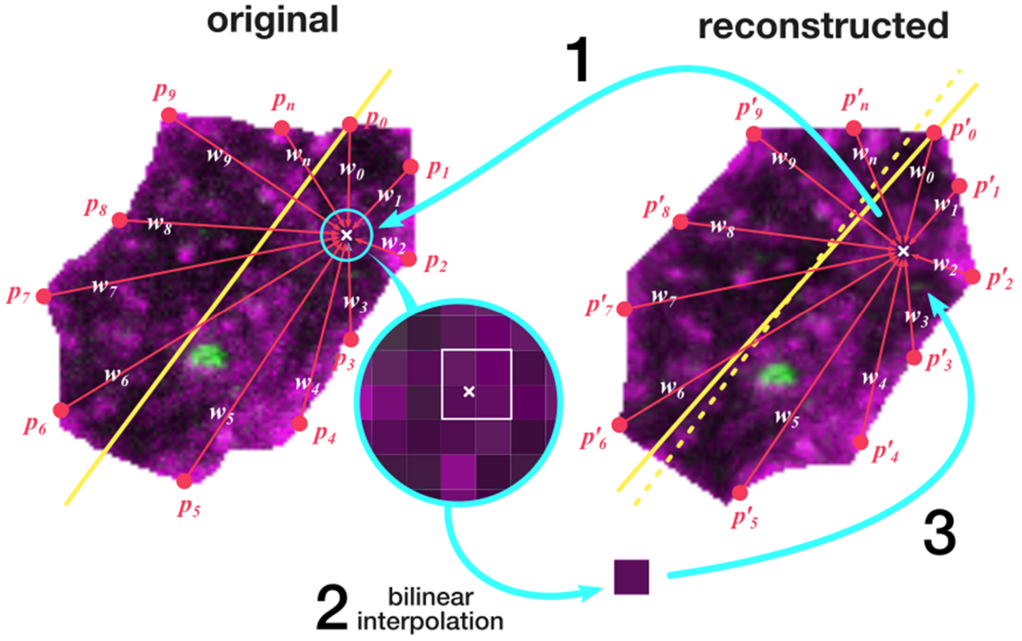

Supplementary figure 2 - Principle of the cell texture morphing*. This particular cell is extracted from the center of the zoomed area in* **Fig. 5***. 1 - Barycentric coordinates of each pixel in the reconstructed cell (white cross on the right panel) are computed from a list of points regularly spaced on the reconstructed contour (p_0', p_1', ..., p_n' : starting from the principal axis). The same weights (w_1, w_2, ...,w_n) are subsequently used to compute a position (white cross on the left panel) relative to a list of points regularly spaced in the original image contour (p_0, p_1, ..., p_n : starting from the principal axis). 2 - a bilinear interpolation of colors is computed from the 4 closest pixels to that subpixelic position. 3 - the pixel in the reconstructed cell (white cross on the right panel) takes this computed color as value. Note that all pixels of the reconstructed SET or any random SET can be recovered this way and particularly the position of any organelles such as the centrioles studied in the results* **Fig. 5***.*

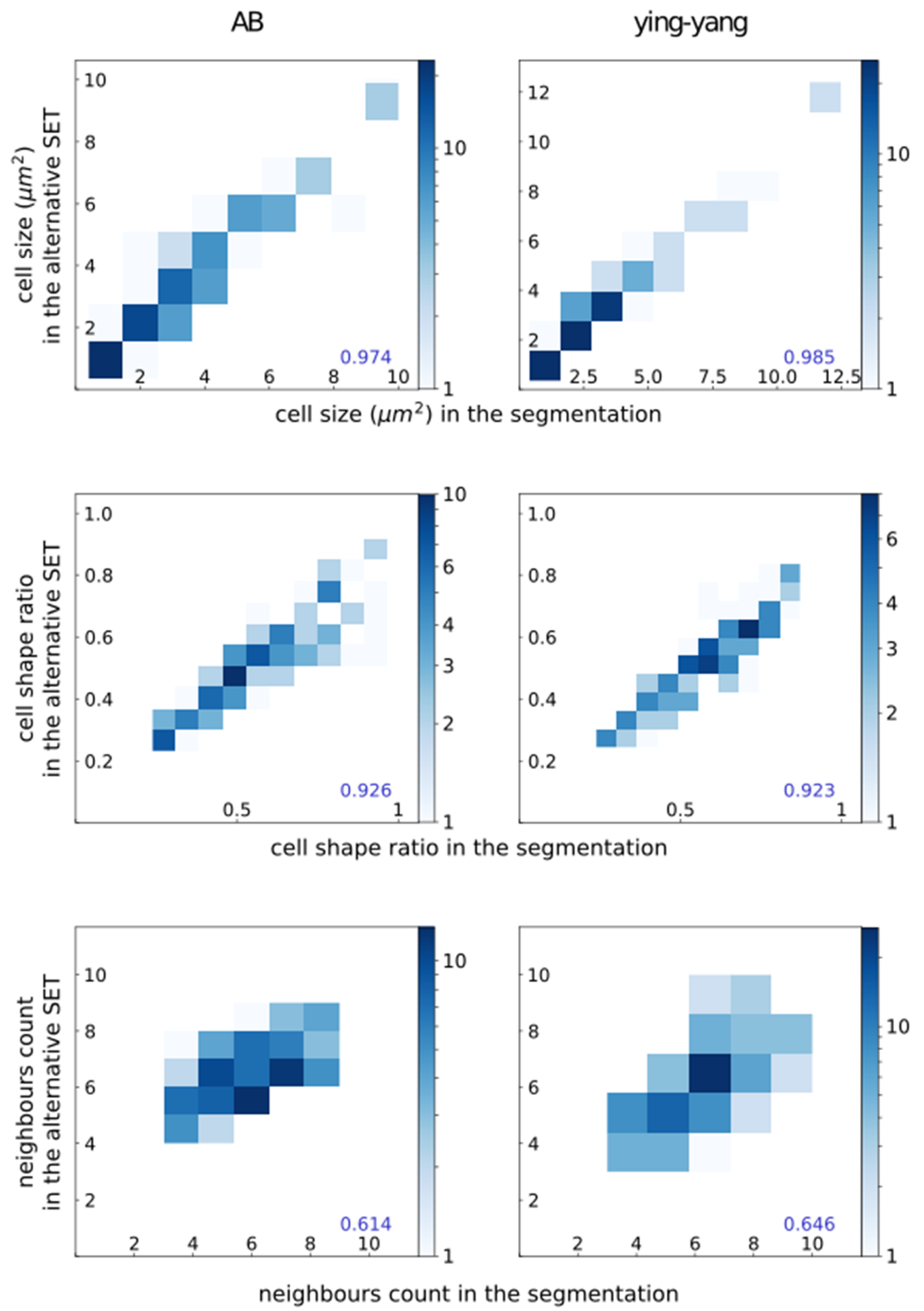

Supplementary figure 3- *Generation of deterministic alternative SET of the P3 ependymal tissue (***Fig. 2****A) preserves single cell size and shape ratio while expectedly breaks local cell organization. Left column: AB image, right column Ying Yang image.*

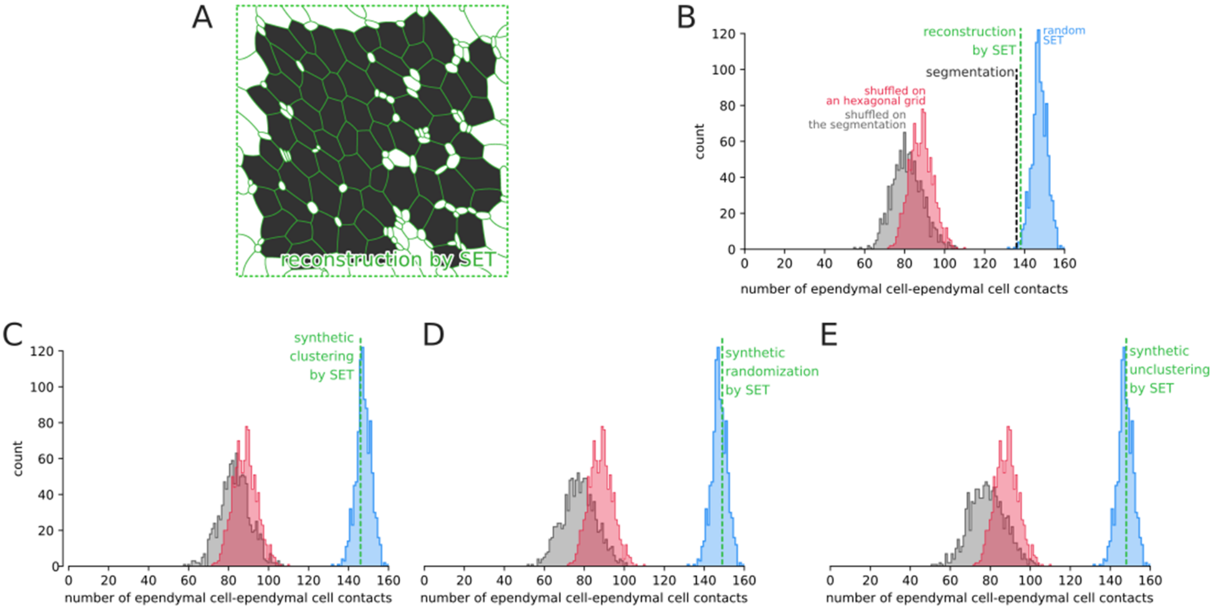

Supplementary figure 4*- Ependymal cells organization at rostral position of the lateral ventricles, comparison with other approaches. A-Reconstruction of* **Fig. 3***A using SET, ependymal cells in grey.* ***B****-Count of the ependymal cell to ependymal cell contacts in the reconstruction by SET panel A (dotted green), in the segmentation panel* **Fig. 3***A (dotted black) and null distributions of the same feature as generated with 3 methods including ours: SET where cells are randomly moved 1000 times (in blue), a model where stem cells are shuffled over a hexagonal grid (in red) and a model where stem cells are shuffled over the actual segmentation (in black) (Methods and* **Supplementary Fig. 6***). The P-value of the reconstruction by SET relative to the null distribution obtained with a 1000 random SET is 0.009.* ***C,D,E****: Evaluation of the 3 models against known synthesized patterns all produced from image* **Fig. 3***A shown in* **Fig. 3***DEF. Our approach by random SET cannot reject the null hypothesis (dotted green line inside our null distribution) in C (Synthesized clustered stem cells), D (Synthesized randomly introduced stem cells) and E conditions (Synthesized repulsed stem cells (anti-clustering)), the two other models consider ependymal pattern present in respectively* **Fig. 3***D,* **Fig. 3***E,* **Fig. 3***F as clustered (ependymal cells would attract each other) while there isn't rule on ependymal cell position.*

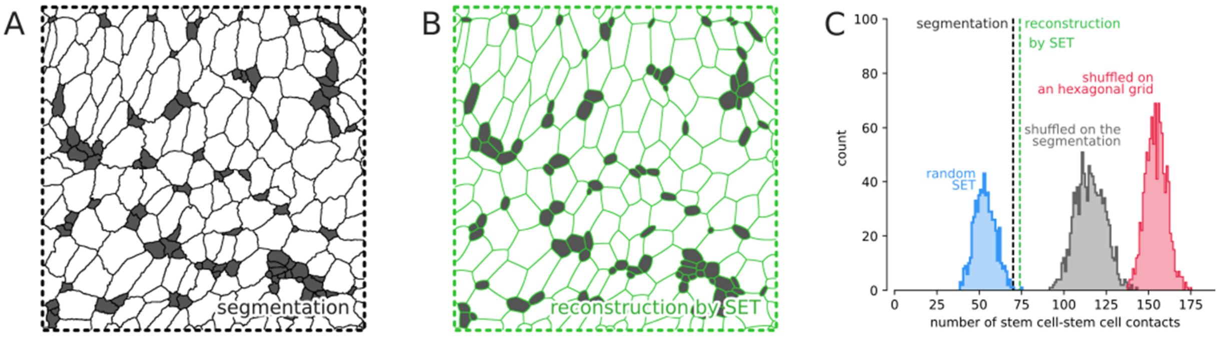

Supplementary figure 5**- **Stem cells are organized in clusters at the rostral position of the lateral ventricles, replicate.** **A-** the image segmentation with stem cells colored in grey of a second individual, approximately at the same ventricular position as the image* **Fig. 3A.* ***B***- *Reconstruction of A using SET, stem cells in grey.* ***C***- *Count of the stem cell to stem cell contacts in the reconstruction by SET panel B (dotted green line), in the segmentation panel A (dotted black line) and null distributions of the same feature as generated with 3 methods including ours: SET where cells are randomly moved 1000 times (in blue), a model where stem cells are shuffled over a hexagonal grid (in red) and a model where stem cells are shuffled over the actual segmentation (in black) (Methods and* **Supplementary Fig. 6***). The P-value of the reconstruction by SET relative to the null distribution obtained with a 1000 random SET is 0.001.*

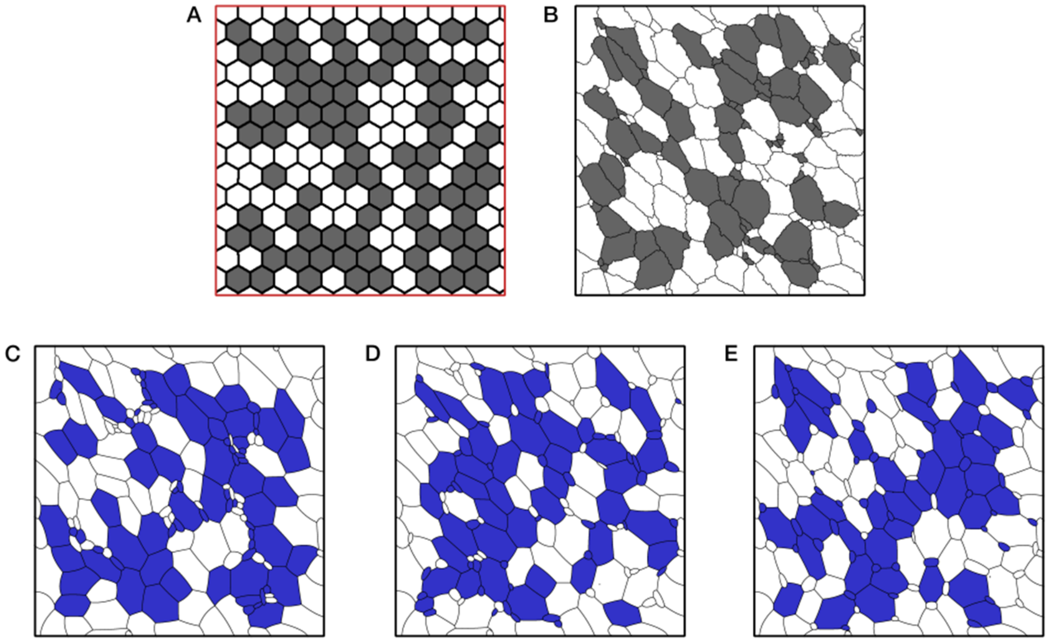

Supplementary figure 6 *- Random synthetic patterns generated with two other methods to produce the null distributions displayed in* **Fig. 3***. A- method 1: random cell fate identity in a honeycomb grid restrained to a cell number (195) close to the number in the segmentation (190). B- method 2: random cell fate identity performed directly in the cell segmentation. C- random cell fate identity performed in the cell segmentation of the clustered cells image (***Fig. 3***D,E). D- random cell fate identity performed in the cell segmentation of the random cells image (***Fig. 3***F,G). E- random cell fate identity performed in the cell segmentation of the repealed cells image (***Fig. 3***H, I). Blue or gray: stem cells, white: ependymal cells*

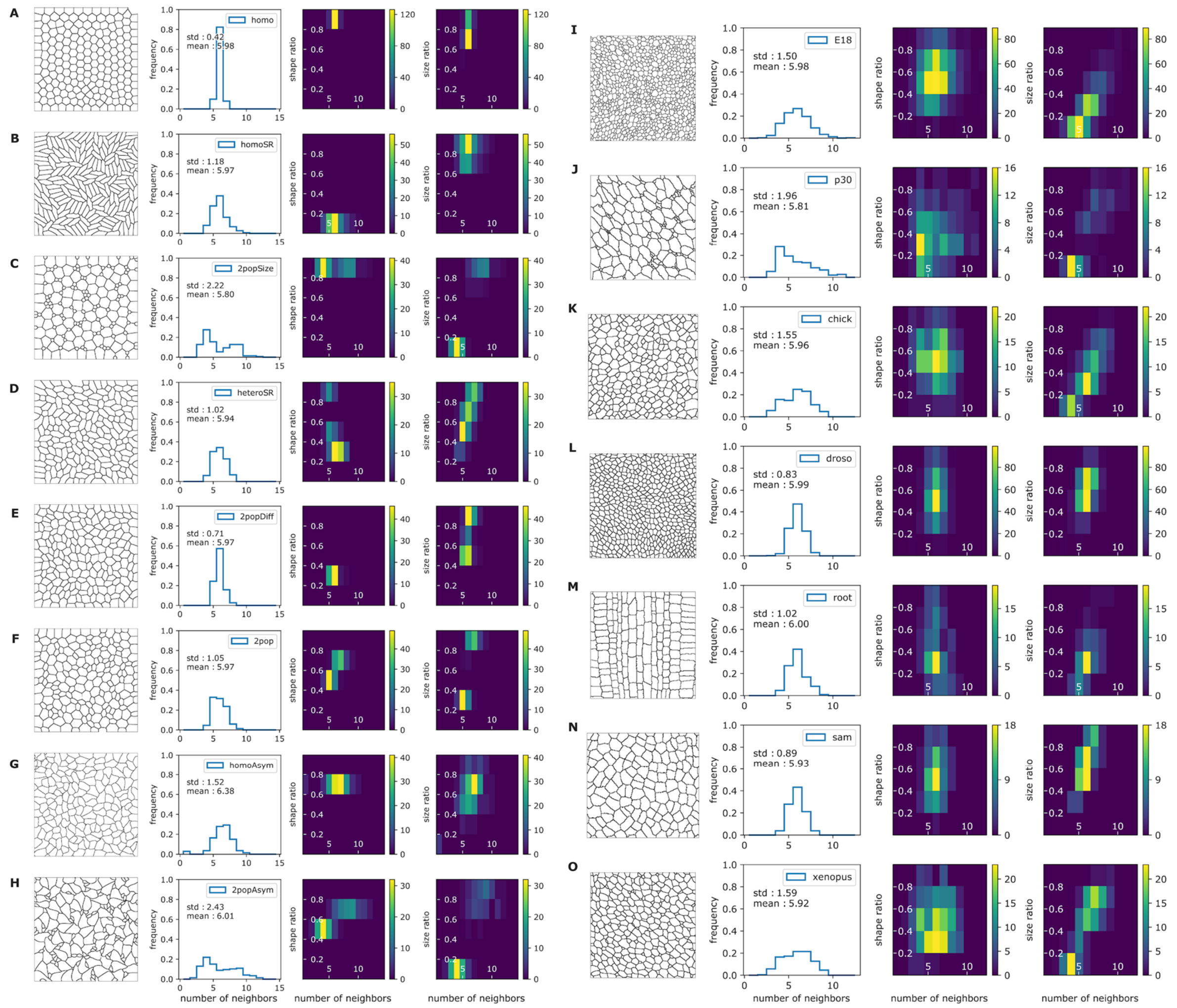

***Supplementary figure 7**- **Impact of tissue cell shape heterogeneity on the cell contact distribution is hardly modelable. A-H -** synthetic SET image with controlled parameters. **I-O** : segmentation of all the real example images shown in the paper. **A-** constant homogenous cell shapes, shape ratio = 1 and constant cell size, no cell asymmetry. **B-** constant homogenous cell shapes, shape ratio : 10, constant cell size. **C-** heterogeneous cell shapes, 2 subpopulations, shape ratio = 1, size ratio = 1/10, no asymmetry. **D-** heterogeneous cell shapes, random shape ratio between 1 and 4, no asymmetry. **E-** heterogeneous cell shapes, 2 populations with different shape ratio (3 and 2), no asymmetry. **F-** heterogeneous cell shapes, 2 populations with different shape ratios (2.13 and 1.55), no asymmetry. **G-** homogeneous cell shapes, shape ratio = 2.13, with asymmetric shapes. **H-** heterogeneous cell shapes, 2 populations with different shape ratios (2.13 and 1.55) and with different asymmetry parameters. **I-** Mouse E18 ependymal tissue used in* **Fig. 5**. ***J-** Mouse P30 ependymal tissue used in* **Fig. 1**, **Fig. 2** *and* **Fig. 3**. ***K-** Chick basilar papilla. **L-** Drosophila melanogaster dorso thorax. **M-** Arabidopsis thaliana root. **N-** Arabidopsis thaliana shoot apical meristem. **O-** Xenopus larva epithelium.*

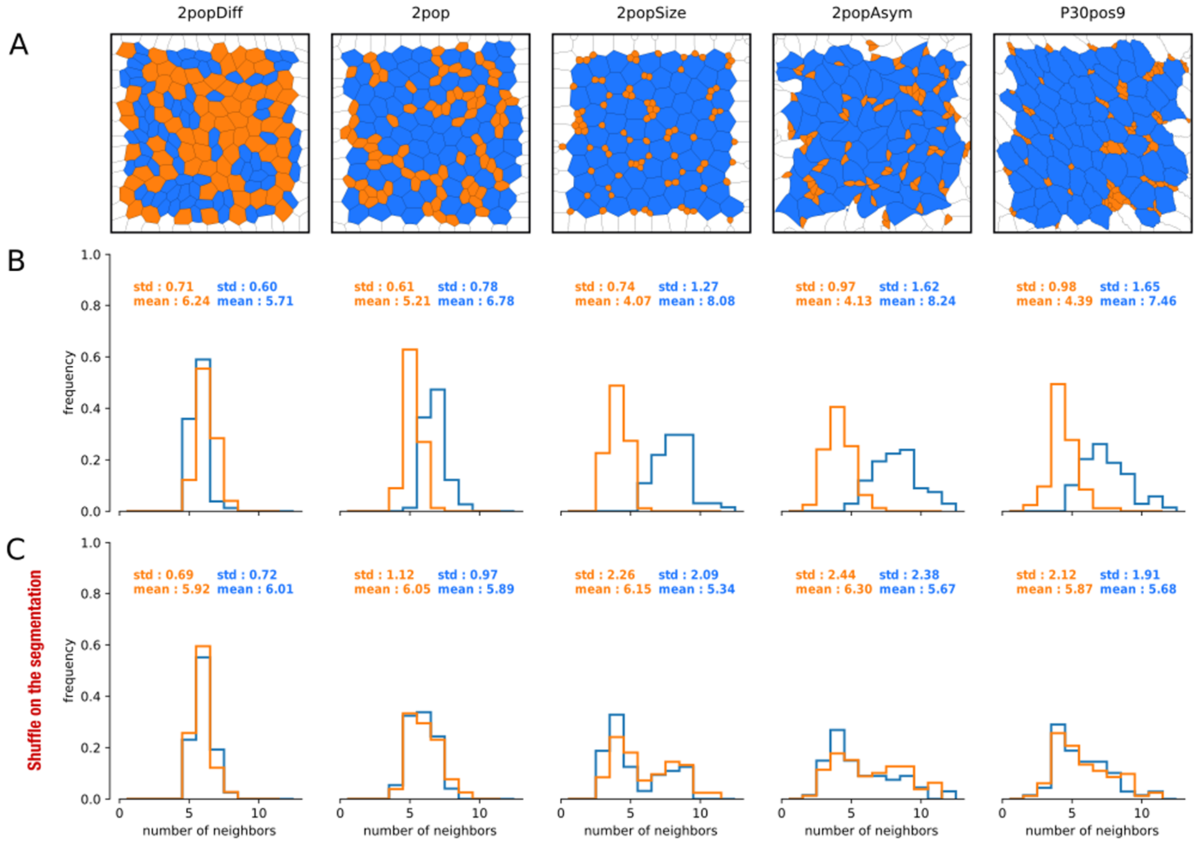

Supplementary figure 8**- **Impact of the 'shuffle on the segmentation' method on the cell neighbor count distribution. A- *examples of tissues with 2 different populations (orange and blue): 4 SET synthetic images and the Mus musculus adult ependymal tissue (P30pos9). 2popDiff : 2 populations of elliptic cells, with different shape ratios (3 and 2). 2pop : 2 populations of elliptic cells, with different shape ratios (2.13 and 1.55) 2popSize: 2 populations of spherical cells of different sizes, size ratio = 1/10. 2pop asym: 2 cell populations with different size, shape ratio and asymmetric shape.* ***B-*** *distributions of the number of neighbors giving the populations shown in images A.* ***C-*** *distributions of the number of neighbors giving a shuffling of the cells of each population shown in images A.*

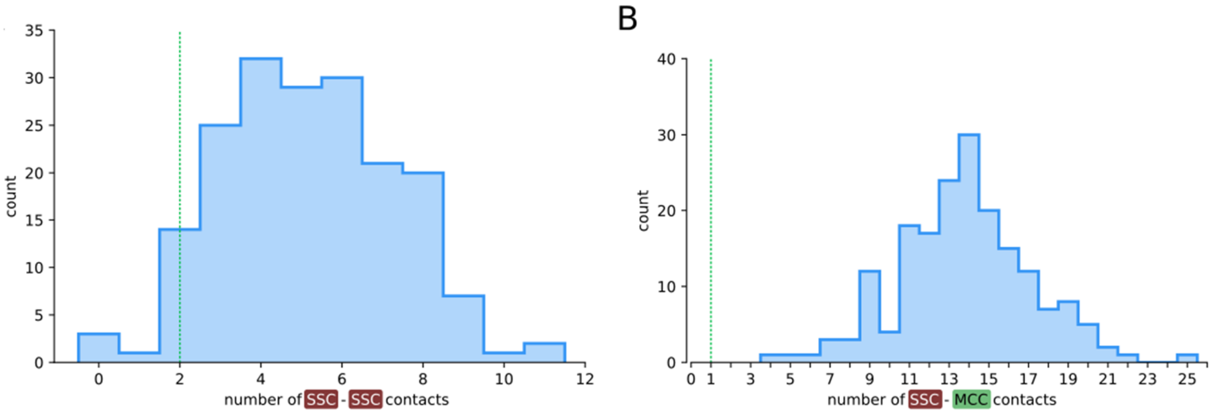

Supplementary figure 9: Spatial organization of small secretory cells (SSC) at st33 Xenopus epidermis. Alternative runs with intercalating cell shuffling. *Blue histogram: null distribution of the number of contacts obtained from the image (***Fig. 4****A). with random SET of all the intercalating cells (all cells except goblet cells). Green dotted line: number of contacts obtained in the reconstruction by SET (***Fig. 4****B).* ***A*** *- Impact of the intercalating cell shuffling on the number of contacts between SSC. P-value = 0.056.* ***B*** *- Impact of the intercalating cell shuffling on the number of contacts between multiciliated cells (MCC) and small secretory cells (SSC). P-value < 0.005.*

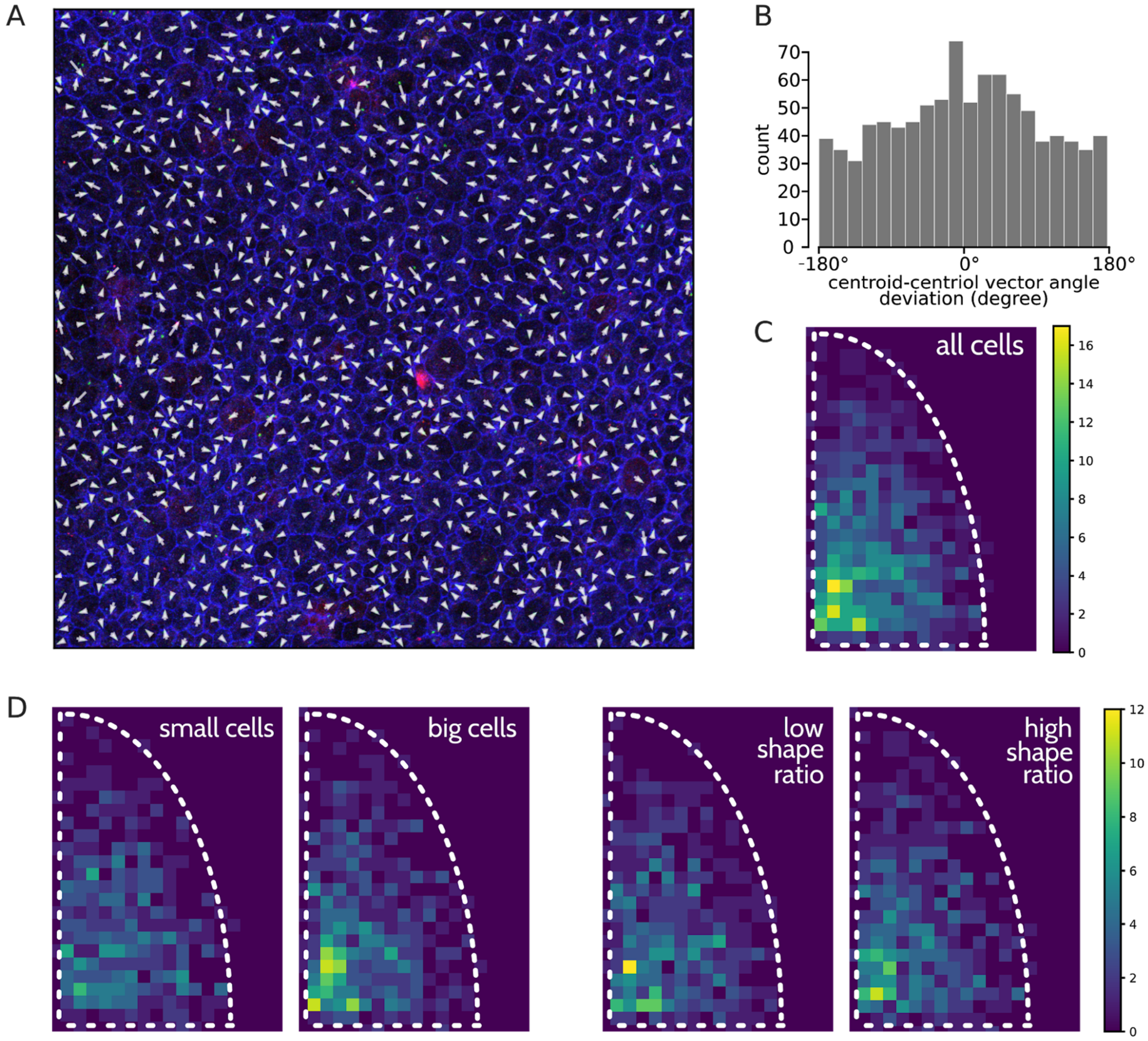

E

		Random SET 1	Random SET 2	Random SET 3	Random SET 4	Random SET 5
Rank 1	mean	89.41	90.29	91.46	88.90	90.87
	std	0.7469	0.7538	0.7536	0.7655	0.7564
	P-value	2.681e-29	9.029e-35	1.287e-43	6.675e-25	9.185e-39
Rank 2	mean	90.33	91.01	90.91	89.26	89.72
	std	0.5160	0.5160	0.5089	0.5121	0.5180
	P-value	5.250e-23	5.210e-29	7.746e-29	3.690e-15	4.145e-18
Rank 3	mean	90.90	90.62	89.84	89.72	89.77
	std	0.4128	0.4133	0.4157	0.4103	0.4088
	P-value	3.678e-24	3.273e-21	4.465e-14	2.007e-13	6.994e-14

Supplementary figure 10 - *Orientation of the centriole of the primary cilium in stem cells of the E18 mice ependyma shown in* **Fig. 5***A, full image.* ***A****- Membrane marked with labeled junction with zo1 (blue) and labeled centrioles with centrin and ninein (green). Overlay with the centroid-centriole vector (white arrow) in each cell.* ***B****- Distribution of the angle deviation from the mean as measured from A.* ***C-****Centriole position*

distribution in the mean quarter cell shape showing that a uniform random sampling could not produce an accurate null distribution for centriole location. ***D-*** *Centriole position distribution in a mean quarter cell shape split in two by cell size on the left and by cell shape ratio on the right. These plots show that the sample distribution could not be used as it is itself related to shape size and shape parameters.* ***E-****examples of p-values obtain when estimating the gaussian parameter of the null distribution from one one random SET only instead of 1000 as in* **Fig. 5***E.*

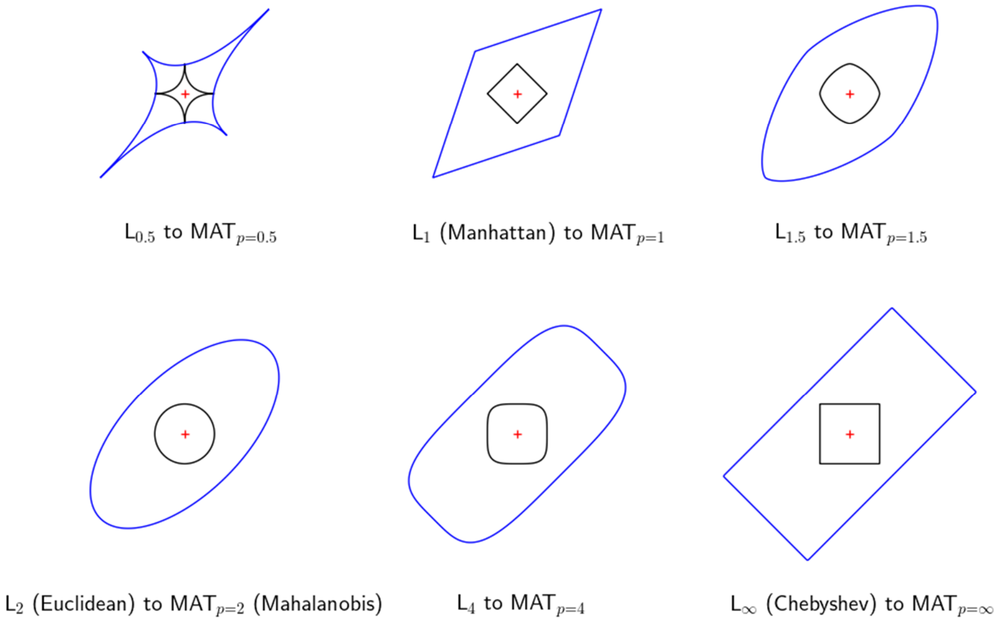

Supplementary figure 11*: 6 examples of the proposed MAT metrics (blue) obtained from rotated and rescaled Minkowski metrics (black). For all examples, both scale factors s1 and s2 (along the x and y axes prior rotation) and the rotation angle alpha are similar and respectively set to the following values: -π/4, 4, 2. All displayed curves are the contour line at MAT distance 1 from the translated origin (red cross) with the specified parameter p.*

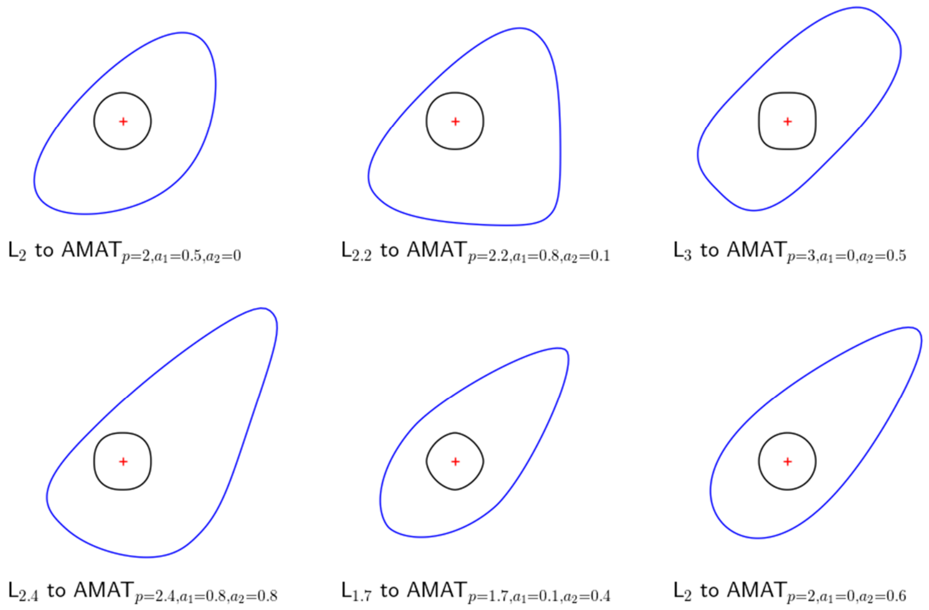

Supplementary figure 12 - *6 examples of the proposed AMAT metrics (blue) obtained from rotated, rescaled and asymmetric transformation of Minkowski metrics (black). For all examples, both scale factors s1 and s2 (along the x and y axes prior rotation) and the rotation angle alpha are similar and respectively set to the following values: -π/4, 4, 2. All displayed curves are the contour line at AMAT distance 1 from the translated origin (red cross) with specified parameters p, a1 and a2.*
