# Supplementary material for: Unraveling spatial cellular pattern by computational tissue shuffling
Source: Commun Biol. 2020 Oct 23;3:605. doi: 10.1038/s42003-020-01323-3 (PMC7584651; doi:10.1038/s42003-020-01323-3)
Supplement: Supplementary file 3 — Description of Additional Supplementary Files [file 42003_2020_1323_MOESM3_ESM.pdf]

Description of Additional Supplementary Files

File Name: Supplementary Movie 1
Description: 2D representation of the fitting process of the level 1 of the AMAT metric to a regular cell contour. Level 0.5 and level 1 are displayed in pink and the main axes in orange and blue.

File Name: Supplementary Movie 2
Description: 2D representation of the fitting process of the level 1 of the AMAT metric to a regular cell contour. Level 0.5 and level 1 are displayed in pink and the main axes in orange and blue.

File Name: Supplementary Movie 3
Description: 2D representation of the fitting process of the level 1 of the AMAT metric to a croissant like cell contour. Level 0.5 and level 1 are displayed in pink and the main axes in orange and blue.

File Name: Supplementary Movie 4
Description: 2D representation of the fitting process of the level 1 of the AMAT metric to a rectangular like cell contour. Level 0.5 and level 1 are displayed in pink and the main axes in orange and blue.

File Name: Supplementary Movie 5
Description: 3D representation of the fitting process of the level 1 of the AMAT metric to a rectangular like cell contour. Level level 1 is displayed in green and the segmented cell contour in red. This representation enables to display the whole distance map around the cell contour with the height corresponding to distance. Note that distances closer than 1 are not displayed as they lie below the surface represented here by the original image.

File Name: Supplementary Movie 6
Description: Representation of the SET iterations using the Llyold-like algorithm to reconstruct the original image in Supplementary Fig. 1 and generate 3 random SET with the same cells.
