# Supplementary material for: Unraveling spatial cellular pattern by computational tissue shuffling
Source: Commun Biol. 2020 Oct 23;3:605. doi: 10.1038/s42003-020-01323-3 (PMC7584651; doi:10.1038/s42003-020-01323-3)
Supplement: Supplementary file 10 — Reporting Summary [file 42003_2020_1323_MOESM10_ESM.pdf]

nature research

Corresponding author(s): Auguste Genovesio & Nathalie Spassky

Last updated by author(s): Sep 21, 2020

Reporting Summary

Nature Research wishes to improve the reproducibility of the work that we publish. This form provides structure for consistency and transparency in reporting. For further information on Nature Research policies, see Authors & Referees and the Editorial Policy Checklist .

Statistics

For all statistical analyses, confirm that the following items are present in the figure legend, table legend, main text, or Methods section.

n/a	Confirmed	
☐	☒	The exact sample size (*n*) for each experimental group/condition, given as a discrete number and unit of measurement
☐	☒	A statement on whether measurements were taken from distinct samples or whether the same sample was measured repeatedly
☐	☒	The statistical test(s) used AND whether they are one- or two-sided<br>*Only common tests should be described solely by name; describe more complex techniques in the Methods section.*
☒	☐	A description of all covariates tested
☐	☒	A description of any assumptions or corrections, such as tests of normality and adjustment for multiple comparisons
☐	☒	A full description of the statistical parameters including central tendency (e.g. means) or other basic estimates (e.g. regression coefficient) AND variation (e.g. standard deviation) or associated estimates of uncertainty (e.g. confidence intervals)
☐	☒	For null hypothesis testing, the test statistic (e.g. *F*, *t*, *r*) with confidence intervals, effect sizes, degrees of freedom and *P* value noted<br>*Give P values as exact values whenever suitable.*
☒	☐	For Bayesian analysis, information on the choice of priors and Markov chain Monte Carlo settings
☒	☐	For hierarchical and complex designs, identification of the appropriate level for tests and full reporting of outcomes
☒	☐	Estimates of effect sizes (e.g. Cohen's *d*, Pearson's *r*), indicating how they were calculated

Our web collection on statistics for biologists contains articles on many of the points above.

Software and code

Policy information about availability of computer code

Data collection	References to the image data are referenced in the manuscript and image samples are made available on the related github repository
Data analysis	The code needed to run the method and a list of examples are made available on the related github repository

For manuscripts utilizing custom algorithms or software that are central to the research but not yet described in published literature, software must be made available to editors/reviewers. We strongly encourage code deposition in a community repository (e.g. GitHub). See the Nature Research guidelines for submitting code & software for further information.

Data

Policy information about availability of data

All manuscripts must include a data availability statement. This statement should provide the following information, where applicable:

- Accession codes, unique identifiers, or web links for publicly available datasets
- A list of figures that have associated raw data
- A description of any restrictions on data availability

The chick image from 38 was provided by David Sprinzak, Guy Richardson and Richard Goodyear and was initially from 39, Copyright 1997 Society for Neuroscience. The Drosophila image from 40 was provided by Yohanns Bellaiche with the permissions of AAAS. The Arabidopsis thaliana root image was provided by Jean-Christophe Palauqui. The Arabidopsis thaliana shoot apical meristem image was provided by Katia Belcram. The xenopus image was provided by Peter Walentek. Mice ependyma images were produced by Nathalie Spassky. A copy of these image data is made available the Github page along the code to run the method https://github.com/biocompibens/cellmodelling.

Field-specific reporting

Please select the one below that is the best fit for your research. If you are not sure, read the appropriate sections before making your selection.

☒ Life sciences ☐ Behavioural & social sciences ☐ Ecological, evolutionary & environmental sciences

For a reference copy of the document with all sections, see nature.com/documents/nr-reporting-summary-flat.pdf

Life sciences study design

All studies must disclose on these points even when the disclosure is negative.

Sample size	Sample size is always 1, the manuscript suggest a way to obtain statistical significance of a pattern using a single image
Data exclusions	no data were excluded
Replication	no replication was performed, the manuscript suggest a way to obtain statistical significance of a pattern using one image
Randomization	random resampling of cell position to build null distributions of cell to cell features were initialized using a uniform distribution
Blinding	randomization was performed using computers and thus blind to the user

Reporting for specific materials, systems and methods

We require information from authors about some types of materials, experimental systems and methods used in many studies. Here, indicate whether each material, system or method listed is relevant to your study. If you are not sure if a list item applies to your research, read the appropriate section before selecting a response.

Materials & experimental systems

n/a	Involved in the study	
☒	☐	Antibodies
☒	☐	Eukaryotic cell lines
☒	☐	Palaeontology
☐	☒	Animals and other organisms
☒	☐	Human research participants
☒	☐	Clinical data

Methods

n/a	Involved in the study	
☒	☐	ChIP-seq
☒	☐	Flow cytometry
☒	☐	MRI-based neuroimaging

Animals and other organisms

Policy information about studies involving animals; ARRIVE guidelines recommended for reporting animal research

Laboratory animals	Genetically engineered mice used in the study are C57/Bl6 background. Both males and females were used for the study. Ages ranging from embryonic day 18 to 2 months old animals were used.
Wild animals	The study does not include any wild animals.
Field-collected samples	The study does not include any field-collected samples.
Ethics oversight	Approval was granted by the french Comité d'éthique en expérimentation animale n°005.

Note that full information on the approval of the study protocol must also be provided in the manuscript.
